# Complete Genome Sequence of *Serratia liquefaciens* Strain ATCC 27592

**DOI:** 10.1128/genomeA.00548-13

**Published:** 2013-08-15

**Authors:** Wayne L. Nicholson, Michael T. Leonard, Patricia Fajardo-Cavazos, Nedka Panayotova, William G. Farmerie, Eric W. Triplett, Andrew C. Schuerger

**Affiliations:** Department of Microbiology and Cell Science, University of Florida, Merritt Island, Florida, USAa; Interdisciplinary Center for Biotechnology Research, University of Florida, Gainesville, Florida, USAb; Department of Plant Pathology, University of Florida, Merritt Island, Florida, USAc

## Abstract

We report the complete genome sequence of *Serratia liquefaciens* strain ATCC 27592, which was previously identified as capable of growth under low-pressure conditions. To the best of our knowledge, this is the first announcement of the complete genome sequence of an *S. liquefaciens* strain.

## GENOME ANNOUNCEMENT

In a previous communication, it was reported that *Serratia liquefaciens* strain ATCC 27592 was able to grow under a combination of low-temperature (0°C), low-pressure (0.7 kPa), and CO_2_-enriched anoxic conditions that were intended to simulate the atmosphere of Mars (1). As part of an effort to further investigate the molecular basis of this response, we report here the complete genome sequence of the strain.

*S. liquefaciens* strain ATCC 27592 was obtained from the American Type Culture Collection (Manassas, VA), and its genome was sequenced at the University of Florida Interdisciplinary Center for Biotechnology Research (UF-ICBR) using the PacBio SMRT system (Pacific Biosciences, Menlo Park, CA). A total of 131,208 reads were obtained, with a mean read length of 4,015 bp. The initial PacBio reads were error corrected using the PacBio RS_PreAssembler.1 module with a minimum subread length of 500 bp, a minimum read quality of 0.80, and a minimum seed read length of 5,000 bp. The error correction process yielded 20,974 reads, with an average length of 5,120 bp. A single scaffold was assembled directly from the error-corrected reads using Celera assembler (CA) version 7.0 software. The initial genome assembly was further refined using the PacBio RS_Resequencing.1 module with Quiver consensus calling. This process removes sequencing errors that remain in the initial CA assembly and produces the final consensus genome sequence. The chromosome has 5,238,612 bp and an overall G+C content of 55.4%. We detected the presence of one 44,107-bp plasmid in the genome. Open reading frame (ORF) prediction and annotation were performed through the Rapid Annotations using Subsystems Technology (RAST) pipeline (2) using Glimmer (3). Of the 4,779 protein-coding ORFs that are present in the circular chromosome, 4,132 (86%) could be assigned by similarity to known annotated protein functions, while 647 (14%) were assigned to unknown protein functions. In addition, 2,762 ORFs (58%) were assigned to Clusters of Orthologous Groups (COG) categories (4) through the Batch CD-Search tool (5). The rRNAs and tRNAs were identified using the “search_for_RNAs” script developed by Niels Larsen (2) and tRNAscan-SE (6), respectively. By these analyses, 79 tRNAs and 6 rRNA operons, comprising 5S, 16S, and 23S rRNA genes, were detected in the genome.

### Nucleotide sequence accession numbers.

The results of this whole-genome shotgun project have been deposited with GenBank under accession no. CP006252 (chromosome) and CP006253 (plasmid).
